# A single dose of Dengvaxia is associated with transient reduction in dengue infection risk among dengue-naïve children

**DOI:** 10.3389/fimmu.2026.1959704

**Published:** 2026-10-14

**Authors:** Maria Abad Fernandez, Jedas Veronica Daag, Lindsay D. Hein, Kristal An Agrupis, Maria Vinna Crisostomo, Michelle Ylade, Jacqueline Deen, Laura J. White, Lakshmanane Premkumar, Ana Lena Lopez, Aravinda M. de Silva

**Affiliations:** 1 Department of Microbiology and Immunology, University of North Carolina, Chapel Hill, NC, United States; 2 Institute of Child Health and Human Development, University of the Philippines Manila, Manila, Philippines

**Keywords:** breakthrough infection, dengue vaccine, dengue virus, dengue-naïve children, neutralizing antibodies, vaccine immunogenicity, wild-type DENV infection

## Abstract

Dengvaxia^®^ induces antibody responses in dengue virus (DENV)-naïve children, but the durability of these responses and their impact on subsequent wild-type (WT) DENV infection remain unclear. Although previous studies have primarily evaluated vaccine efficacy against dengue disease, the impact of Dengvaxia^®^ on susceptibility to WT DENV infection and subsequent immune responses remains less well defined. We evaluated vaccine-induced immunity and susceptibility to WT DENV infection in a longitudinal cohort of 186 seronegative children, including 114 who received a single dose of Dengvaxia^®^ and 72 who remained unvaccinated, followed for 40 months. Samples collected at baseline and two follow-up time points (20 months [T1] and 40 months [T2]) were analyzed using yellow fever virus (YFV) non-structural protein 1 (NS1) antibody assays, which is expressed by Dengvaxia^®^ but not by WT DENV, enabling discrimination between vaccine-associated and WT infection-induced responses. At T1, 61% of vaccinated children were responders. Additional low-level YFV NS1 antibody responses were detected in some children originally classified as vaccine non-responders, suggesting that the predefined binary classification may not capture the full range of vaccine-associated antibody responses. Low-level YFV NS1 responses remained detectable through T2. During the first follow-up period (baseline-T1), vaccination was associated with a reduced risk of WT DENV infection (vaccine effectiveness [VE]=45%). During the second period (T1-T2), no difference in infection risk was detected between groups. Following breakthrough infection during the first follow-up period, vaccinated children developed antibody responses with greater magnitude and broader serotype breadth than unvaccinated children with primary infection, consistent with vaccine-associated immune priming following WT DENV infection. In contrast, during the second period (T1-T2), differences in antibody magnitude and breadth following infection were no longer detected between groups. Together, these findings suggest that a single dose of Dengvaxia^®^ in DENV-seronegative children was associated with a reduced risk of WT DENV infection and with differences in subsequent antibody responses during the first follow-up period, but these associations were no longer observed during the second period.

## Introduction

Dengue virus (DENV), a mosquito-borne flavivirus with four antigenically distinct serotypes (DENV1–4), causes a broad spectrum of clinical outcomes, ranging from asymptomatic infection to severe, life-threatening disease (1). Globally, an estimated 400 million DENV infections occur annually, predominantly in tropical and subtropical regions (2). Natural infection induces long-lived protection against the infecting serotype; however, subsequent secondary infection with another serotype is associated with increased risk of severe disease, a phenomenon often attributed to antibody-dependent enhancement (ADE) (3). These immunological dynamics pose unique challenges for dengue vaccine development, requiring the induction of both breadth and durability of protective immunity.

Dengvaxia^®^ (CYD-TDV) is a live-attenuated, tetravalent dengue vaccine developed to protect against all four DENV serotypes (4). In 2015, it was approved for use in children aged nine years and older living in areas of high dengue endemicity. However, subsequent analyses of clinical trial data revealed an increased risk of severe dengue and hospitalization among vaccine recipients who were DENV-seronegative at baseline (5). These findings led to revised recommendations restricting vaccine use to individuals with evidence of prior dengue infection (6). Limited efficacy in seronegative children has been attributed to unbalanced replication among the vaccine’s tetravalent components, with the DENV4 strain replicating more efficiently than the others, resulting in predominantly DENV4-specific and cross-reactive antibodies to DENV1–3 that may contribute to enhanced disease risk (7).

Prior to revised recommendations restricting the use of Dengvaxia^®^ to individuals with prior dengue infection, nearly one million children were vaccinated in the Philippines (8). In parallel with the vaccine rollout, we established a 5-year prospective pediatric cohort in Cebu to assess vaccine immunogenicity and efficacy by baseline dengue serostatus (9). We previously reported vaccine efficacy against virologically confirmed dengue within this cohort (9) and subsequently conducted detailed immunological analyses in a subgroup of 138 children who were DENV-seronegative at baseline (BL), focusing on individual-level vaccine responses and their relationship to safety and efficacy (10). At 20 months of follow-up, 38% of these baseline seronegative vaccinated children lacked detectable DENV-specific antibody responses by standard assays and were classified as vaccine “non-responders” according to the predefined study criteria (10).

Vaccine non-responders are routinely included in efficacy studies and immunogenicity analyses evaluating seroconversion and the heterogeneity of vaccine-induced immune responses (5, 11, 12). However, the absence of detectable antibodies does not necessarily indicate an absence of vaccine-induced immune priming (13–15). In vaccines such as influenza and hepatitis B, post-exposure or booster responses have revealed underlying immune priming despite low or undetectable circulating antibody responses (14, 15). In dengue vaccination, however, immunogenicity assessments have largely relied on circulating neutralizing antibodies (NAbs), potentially overlooking low-level antibody responses that may not be captured by standard assays (7). These limitations highlight the need to better define the spectrum, durability, and characteristics of vaccine-induced immune responses in dengue-naïve children, as well as how these responses are associated with susceptibility to subsequent WT DENV infection.

To better understand the durability and characteristics of antibody responses stimulated by Dengvaxia^®^ in dengue-naïve children and their association with susceptibility to WT DENV infection, we addressed three interrelated questions in this study. First, to better define vaccine responders and individuals with low or undetectable responses by standard assays, we used an enhanced detection approach incorporating YFV NS1 antibody measurements at 20 and 40 months after vaccination (T1 and T2). Second, we evaluated antibody responses following breakthrough WT DENV infection by characterizing DENV-specific antibody magnitude and serotype breadth during the two follow-up periods. Finally, we examined how these immunological patterns related to susceptibility to WT DENV infection during the first (baseline to T1) and second (T1 to T2) study periods.

## Methods

### Cebu cohort study and ethics statement

Between 2016 and 2017, 2,996 children aged 9–14 years were enrolled in a longitudinal cohort study. Of these, 1,782 children received a single dose of Dengvaxia^®^ during a mass vaccination campaign, while 1,214 remained unvaccinated. Demographic information and serum samples were collected from all participants at baseline, prior to vaccination. Baseline dengue immune status was assessed using a PanBio indirect IgG ELISA and DENV1–4 standard neutralization assays (two dilutions, 1:40 and 1:200), and 241 children were classified as dengue-naïve, including 148 vaccinated and 93 unvaccinated participants (16). In this cohort, the PanBio IgG ELISA showed 95.2% sensitivity and 93.4% specificity relative to FRNT for distinguishing DENV-seropositive from DENV-seronegative children (16).

For the present analysis, we focused on a subset of dengue-naïve children from the previously described cohort (10) for whom serum samples were available at baseline and at two follow-up time points, approximately 20 months (T1) and 40 months (T2) post-baseline. This analytic subset included 186 children (114 vaccinated and 72 unvaccinated), reflecting those with complete longitudinal sampling through T2. The remaining DENV-naïve children were not included because complete longitudinal serum samples through T2 were unavailable. Sex was recorded for all participants and both males and females were included in the study. Sex was not considered as a biological variable in the analyses. Baseline samples were collected prior to vaccination, which occurred approximately two months after enrollment. Follow-up sampling occurred within predefined windows that varied slightly between participants; therefore, time points are reported relative to baseline throughout the manuscript.

The study protocol was approved by the University of Manila Research Ethics Board (UPM-REB 2016-435-01) and was registered at ClinicalTrials.gov (NCT03465254). Parents or guardians provided written informed consent and children provided documented oral assent before participating in the study, in accordance with local regulations.

### Cells and virus

Vero cells (CCL-81, ATCC) and furin-overexpressing Vero cells generously received from Dr. Ralph Baric were cultured as monolayers at 37 °C with 5% CO2 in Opti-Pro serum-free medium (Invitrogen), supplemented with 10% fetal bovine serum (FBS) and 4 mM glutamine (Gibco). DENV1–4 strains that match circulating genotypes in the Philippines were produced as previously described (17, 18). DENV virus strains used were: DENV1 (2014 Sri Lanka strain, GenBank accession no. KJ726662), DENV2 (2016 Sri Lanka strain, MK579857), DENV3 (1986 Sri Lanka strain, JQ411814) and DENV4 (1992 Sri Lanka strain, KJ160504).

### Focus reduction neutralization test

Neutralization assays were conducted following previously described procedures (17, 18). In brief, 1.7x10^4^ Vero cells per well were plated one day before infection and incubated overnight at 37 °C. Human immune sera were serially diluted four-fold and mixed with low-passage clinical isolates of DENV1-4, produced in Vero-furin cells. The serum-virus mixtures were incubated at 37 °C for one hour before being added to confluent cells. After an additional hour, a 1% methylcellulose overlay was applied, and the cells were incubated for 2–3 days. The cells were then fixed with 80% methanol, blocked with 5% non-fat dried milk, and immunostained using mouse 4G2 and 2H2 anti-pan flavivirus monoclonal antibodies. Secondary detection was carried out with a horseradish peroxidase (HRP)-labeled goat anti-mouse IgG antibody (Jackson ImmunoResearch Laboratories), and HRP activity was visualized with the TrueBlue substrate. Images of all wells were captured using the Cellular Technology Limited (CTL) imaging system and ImmunoSpot software, and foci were quantified with the Viridot plaque counter in R. Serum neutralization titers were determined by calculating the serum dilution resulting in a 50% reduction in foci relative to control wells with virus without serum.

### Luminex-based assays to measure binding antibodies to NS1 and EDIII

Binding antibodies to DENV envelope protein domain 3 (ED3) and nonstructural protein 1 (NS1) of DENV1–4, as well as YFV NS1, were measured in unvaccinated and vaccinated children using a Luminex-based multiplex assay, as previously described (19). Briefly, biotinylated ED3 antigens and biotinylated bovine serum albumin (BSA) were coupled to unique MagPlex^®^-Avidin Microspheres (Luminex), while His-tagged NS1 antigens (The Native Antigen Company) were immobilized onto avidin-coated microspheres via anti-His tag antibody (Abcam). ED3-, NS1-, and BSA-conjugated microspheres were mixed in equal ratios and plated at 2,500 beads per antigen in 50 µL/well on 96-well plates. Human serum samples were diluted 1:500 for all antigens; in addition, a 1:50 dilution was used for YFV NS1 to enhance detection of low-level antibody responses. Diluted sera were incubated with the antigen-coupled microspheres for 1 hour at 37 °C and 700 rpm, followed by incubation with goat anti-human IgG Fc multi-species SP ads-PE (Southern Biotech) after three washes. Antibody responses were measured using a Luminex 200 analyzer and expressed as median fluorescence intensity (MFI), with non-specific binding to BSA subtracted.

For measurements at the 1:500 dilution (all antigens), a seropositivity threshold was applied to facilitate interpretation across individuals. The threshold was calculated from MFI values measured in 136 DENV-naïve children, taking the mean plus three standard deviations (mean + 3 SD). A sample was considered positive if its MFI exceeded this threshold and showed a ≥2-fold increase from baseline to T1.

For measurements at 1:50 dilution, YFV NS1 antibody responses were defined based on the fold-increase in Luminex signal between baseline and T1 (T1/BL ratio). Receiver operating characteristic (ROC) analysis comparing originally classified vaccine responders (n=52) and unvaccinated children who remained free of WT DENV infection during the BL-T1 interval (n=41) was performed to determine the optimal threshold for discrimination between these two groups. The area under the curve (AUC) was 0.94 (95% CI 0.89–0.99), and the Youden index identified a 3.55-fold increase as the optimal cutoff within this dataset. For consistency with conventional serologic criteria and to avoid relying on a threshold optimized within the study population, a ≥4-fold increase was applied to classify YFV-specific vaccine responses in all subsequent analyses, yielding 82.7% sensitivity and 92.7% specificity relative to these predefined reference groups. Sensitivity analyses using the ROC-derived 3.55-fold threshold yielded similar results.

### Definition of vaccine response, non-response and DENV infection

Vaccine responders and non-responders were initially classified based on a composite immunological profile integrating multiple assays. Children were considered vaccine responders if they showed YFV NS1 antibody positivity by ELISA and/or Luminex at 1:500, or if they seroconverted to DENV-ED3 antigens without seroconversion to DENV-NS1 antigens. Non-responders were defined as those lacking detectable responses across these criteria. To enhance detection of low-level YFV NS1 responses, children who remained free of breakthrough WT DENV infection through T1 were additionally assessed using Luminex at a 1:50 dilution.

DENV infection was defined as previously described based on serological evidence of seroconversion to DENV NS1 and/or serotype-specific antibody profiles in longitudinal paired samples collected over time (10). For infections identified during the BL-T1 interval, the infecting serotype was inferred from post-infection neutralization profiles based on seroconversion to a single DENV serotype or a ≥4-fold higher neutralizing antibody titer to one serotype relative to the other serotypes. Infections for which no single serotype met either criterion were classified as indeterminate. Both symptomatic and asymptomatic infections were included.

### Statistical analysis

Statistical analyses were performed using GraphPad Prism (version 10.1.2), with probabilistic index effect sizes and 95% confidence intervals calculated in R (version 4.3.2). Paired comparisons between baseline and post-vaccination time points were performed using the Wilcoxon signed-rank test. Longitudinal analyses across three time points (baseline, T1, T2) were conducted using the Friedman test with Dunn’s *post-hoc* multiple comparisons. All analyses were two-tailed, and p values <0.05 were considered statistically significant.

Neutralizing antibody titers (NAb) below the assay positivity threshold (<20) were assigned a value of 5 prior to log10 transformation to calculate cumulative titers per individual. Anti-ED3 IgG binding responses measured by Luminex at the 1:500 dilution were assigned a value of 1 for MFIs below the assay positivity threshold (MFI < 1237) to include low-level responses in the analysis. Cumulative titers and binding responses were compared across groups using the Kruskal-Wallis with Dunn’s *post-hoc* correction. Serotype breadth was defined for each individual as the number of DENV serotypes (ST) meeting the assay-specific positivity threshold (range, 0-4ST) and was compared across groups using the Kruskal-Wallis test with Dunn’s *post-hoc* correction. Differences in the categorical distribution of serotype breadth were additionally assessed using Fisher’s exact test. Cumulative antibody magnitude was used to summarize the overall magnitude of antibody response across DENV1–4, whereas serotype breadth captured the number of serotypes with detectable responses. These measures were analyzed as complementary endpoints because responses of similar overall magnitude may differ in the number of serotypes recognized. For comparisons of post-infection antibody responses between vaccinated and unvaccinated children, effect sizes for antibody magnitude and serotype breadth were estimated using the Mann-Whitney probabilistic index, with values >0.5 indicating a greater probability of a higher response in the vaccinated group relative to the unvaccinated reference group. Ties were assigned half-weight, and 95% confidence intervals (CI) were estimated using nonparametric bootstrap resampling within groups. As an exploratory sensitivity analysis, post-infection antibody responses were additionally compared between vaccine responders and unvaccinated children with inferred DENV2 infection, the predominant assignable infecting serotype in both groups.

Risk ratios (RRs) and 95% CIs were calculated to compare infection risk between groups. RRs were estimated using binomial regression models with a log link, with DENV-naïve unvaccinated individuals as the reference group. Vaccine effectiveness (VE) was calculated as VE= (1−RR) × 100. Confidence intervals for RR were derived from model-based standard errors. Analyses were performed separately for each study period. Comparisons addressing the primary study questions were hypothesis-driven, whereas additional subgroup analyses were exploratory.

### Study approval

The study protocol was approved by the University of the Philippines - Manila Research Ethics Board (UPM REB 2016-435-01) and is registered at clinicaltrials.gov (NCT03465254). Parents or guardians provided written informed consent, and children provided documented oral assent before participation in the study, in accordance with local regulations.

## Results

### DENV-naïve children study cohort

**Table 1** summarizes the demographic characteristics of 186 DENV-naïve children (114 vaccinated and 72 unvaccinated) with longitudinal serum samples collected at baseline, month 20 (T1), and month 40 (T2).

**Table 1:** Cohort characteristics for children with longitudinal samples at T1 and T2.

Characteristics	Vaccinees (n=114)	Unvaccinated (n=72)	p-value
Age, years	10 (9-11)	10 (9-11)	0.0605
Male sex, n (%)	53 (47)	35 (49)	0.8803
City Bogo, n (%)	57 (50)	30 (42)	0.3661
Serum collection, T1 (months)	19 (17-21)	21 (18-24)	0.0002
Serum collection, T2 (months)	40 (34-41)	40 (33-41)	0.8479

p-values were calculated using Mann–Whitney U test for continuous variables and Fisher’s exact test for categorical variables. For binary variables (sex, city), only one category is shown; the complementary category is implied; all participants were from either Bogo or Balamban. p value <0.05 was considered statistically significant.

The median age at baseline was comparable between vaccinated and unvaccinated children. The proportions of male participants and children residing in Bogo were comparable between groups. At T1, serum samples from vaccinated children were collected slightly earlier than those from unvaccinated children (median 19 vs. 21 months post-baseline; p=0.0002), whereas no difference in T2 collection times was detected between groups.

### Low-level YFV NS1 Ab responses in children previously designated vaccine “non-responders”

Dengvaxia^®^ is based on the YFV 17D vaccine backbone and expresses the prM and E proteins of each DENV serotype, while all non-structural (NS) proteins are derived from YFV. Accordingly, antibody responses to YFV NS1 have been used to identify antibody responses associated with vaccination and to distinguish them from responses to wild-type (WT) DENV infection. Using a composite immunological classification that included YFV NS1 antibody measurements by Luminex at 1:500 serum dilution and IgG ELISA, we previously classified 32% of vaccinated children as “non-responders” at T1 (10).

To investigate whether low-level YFV NS1 antibody responses could be detected in children previously classified as non-responders, we re-analyzed samples from vaccinated children previously classified as responders (n=52) or non-responders (n=35) using the Luminex assay at 1:50 dilution. Antibody responses were evaluated as fold change relative to baseline to account for inter-assay variability and differences in background signal. Unvaccinated children (n=41) who remained free of WT DENV infection over the same interval were included as negative controls to empirically define response thresholds.

Using a ≥4-fold increase in YFV NS1 antibody signal between baseline and T1, 83% (43/52) of children previously classified as vaccine responders met this response criterion, compared with 37% (13/35) previously classified as vaccine non-responders and 7% (3/41) of unvaccinated children (**Figure 1A**).

**Figure 1:**
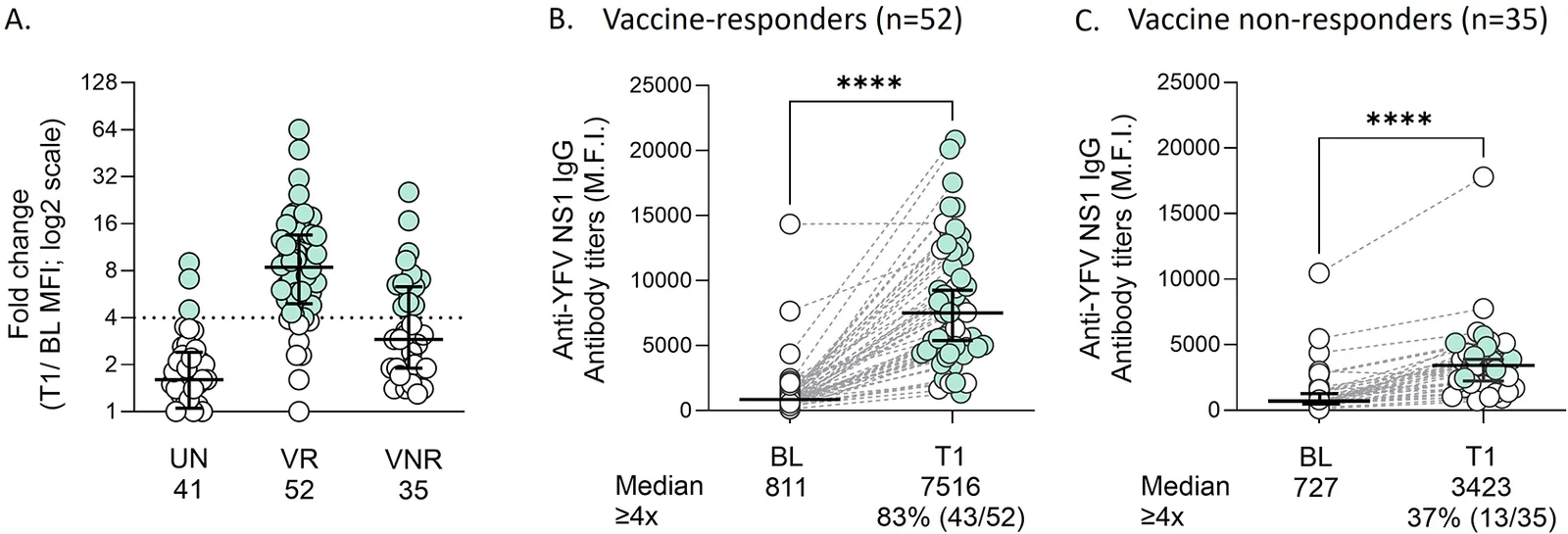
Detection of low-level YFV NS1 antibody responses using the Luminex assay at 1:50 serum dilution. **(A)** Fold change in anti-YFV NS1 IgG MFI at T1 relative to BL in vaccinated children previously classified as responders (VR; n = 52) or non-responders (VNR; n = 35), and in unvaccinated controls (UN; n = 41) who remained free of WT DENV infection over the BL-T1 interval. Each point represents an individual participant. Horizontal lines indicate group medians with interquartile ranges. The dashed line denotes the ≥4-fold response criterion used in subsequent analyses. **(B)** Raw anti-YFV NS1 IgG MFI values at BL and T1 in vaccine responders (n = 52) and **(C)** in children previously classified as vaccine non-responders (n = 35). Lines connect paired samples from the same individual. Medians with interquartile ranges are indicated. Across all panels, colored symbols indicate individuals with a ≥4-fold increase relative to baseline. Statistical analysis was performed using Friedman tests with Dunn’s *post hoc* multiple comparisons. Significant differences between time points are denoted as ****p < 0.0001; ***p < 0.001; **p < 0.01.

Consistent with the fold-change analysis, raw median fluorescence intensity (MFI) values were similar at baseline between children previously classified as vaccine responders and non-responders (median 811 vs. 727 MFI, respectively; p=0.721), whereas at T1, YFV NS1 MFI was higher among responders than non-responders (median 7, 515.5 vs. 3,423 MFI; p < 0.001). YFV NS1 antibody signal increased significantly from baseline to T1 within both groups (**Figures 1B, C**).

To assess the durability of vaccine-induced responses, we analyzed longitudinal sera collected at baseline, T1 and T2 from vaccinated children without breakthrough WT DENV infection through T2. This subset included 23 vaccine responders and 12 children previously classified as non-responders. Among individuals meeting the ≥4-fold response criterion at T1 (n=19 responders; n=4 non-responders), 84% (16/19) of responders and 75% (3/4) of non-responders maintained a ≥4-fold increase at T2, indicating persistence of detectable YFV NS1 antibody responses over time (**Figures 2A, B**).

**Figure 2:**
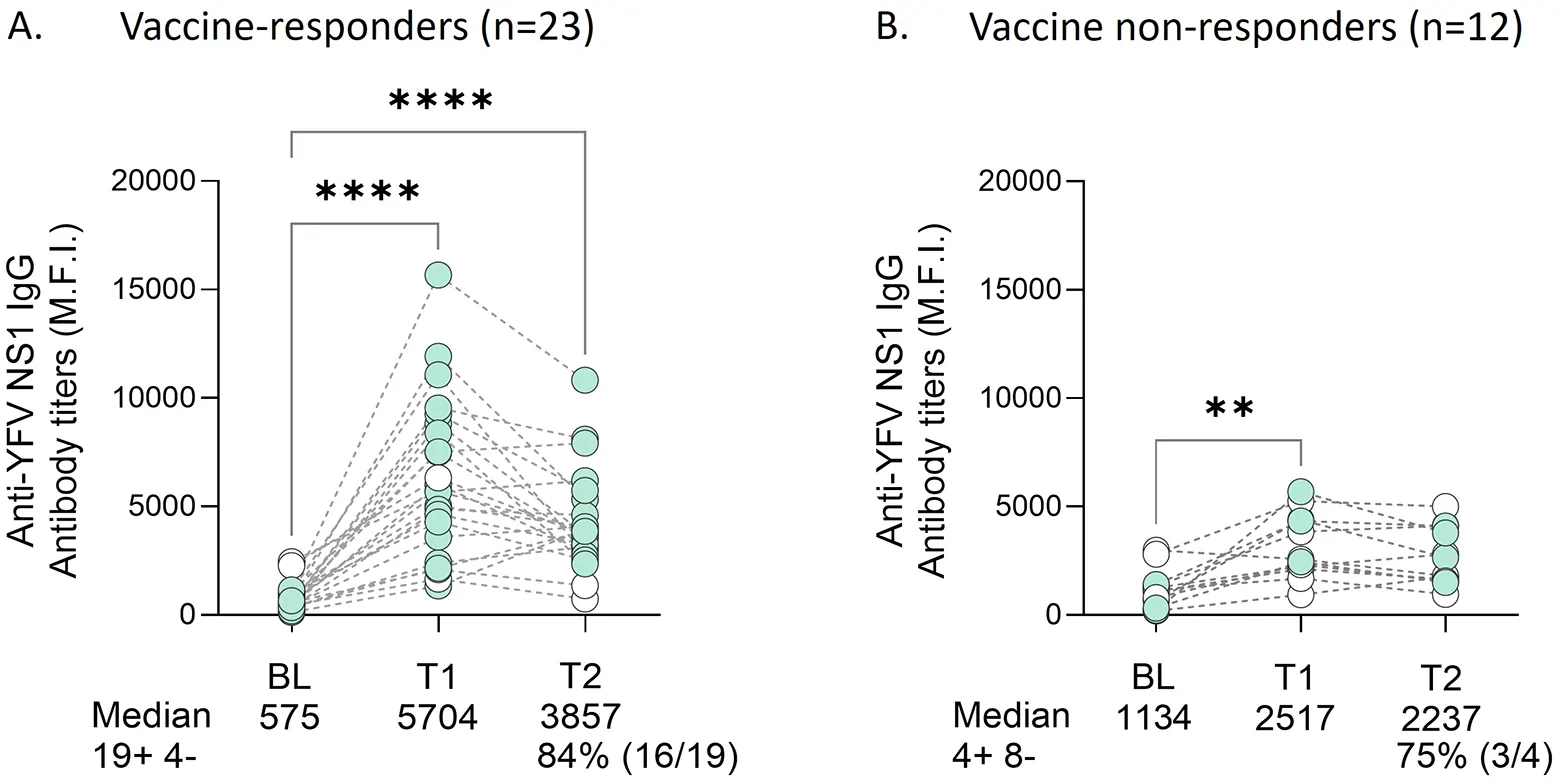
Longitudinal YFV NS1 antibody responses in vaccinated children without breakthrough WT DENV infection through T2. **(A)** Vaccine responders (n=23) and **(B)** previously classified non-responders (n=12) who remained free of WT DENV infection through T2. Lines connect individual children over time. Colored symbols indicate a ≥4-fold increase relative to baseline. Among individuals meeting the ≥4-fold response criterion at T1, 84% (16/19) of responders and 75% (3/4) of non-responders maintained a ≥4-fold increase at T2. Statistical analysis was performed using the Friedman test with Dunn’s *post hoc* multiple comparisons. Significant differences between timepoints are denoted as ****p < 0.0001; ***p < 0.001; **p < 0.01.

Accordingly, subsequent analyses retained the original responder classification unless otherwise stated. The 1:50 YFV NS1 assay was used to identify low-level antibody responses detectable at T1 in vaccinated children without breakthrough WT DENV infection. It was not used to retrospectively redefine responder status across the cohort, as it does not capture vaccine-induced responses that may have occurred earlier and subsequently declined before T1.

### Vaccine response profiles are associated with susceptibility to DENV infection

To assess whether vaccine-associated antibody responses were associated with susceptibility to subsequent WT DENV infection, we compared infection rates among unvaccinated children, vaccine responders, and vaccine non-responders during the first and second study periods.

During the first study period (BL–T1), unvaccinated children experienced the highest incidence of WT DENV infection, with 43% becoming infected. In comparison, lower infection rates were observed among vaccinated children, including both vaccine responders (25%) and non-responders (22%). Relative to unvaccinated children, vaccine responders showed reduced infection risk (risk ratio (RR)=0.57, 95% CI = 0.34–0.92), corresponding to an estimated vaccine effectiveness (VE) of approximately 43%. Vaccine non-responders showed a similar reduction in infection risk (RR = 0.52, 95% CI = 0.26–0.90; VE = 48%) (**Table 2**). Thus, vaccination was associated with a lower susceptibility to WT DENV infection during the first follow-up period in both responder and non-responder groups. In contrast, during the second study period (T1–T2; approximately months 20 to 40 of follow-up), vaccination status was no longer associated with reduced risk of WT DENV infection. Prior natural DENV infection was associated with a 76% reduction in infection risk compared with dengue-naïve children (RR = 0.24, 95% CI = 0.08–0.55). Among vaccinated children, infection risk did not differ according to neutralizing antibody profiles or vaccine responder status at T1. Similarly, vaccine non-responders showed no evidence of a lower infection risk, regardless of detectable vaccine-associated antibody responses (**Table 2**).

**Table 2:** Risk of wild-type DENV infection during the first (BL–T1) and second (T1–T2) study periods across vaccination and immunological subgroups.

BL-T1				T1-T2			
Groups (N)	Infections n (%)	RR (95% CI)	VE (%)	Groups (N)	Infections n (%)	RR (95% CI)	VE (%)
UN (72)	31 (43)	reference	–	DENV naive (41)	22 (54)	reference	–
				DENV infected (31)	4 (13)	0.24 (0.08-0.55)	76
VR (69)	17 (25)	0.57 (0.34-0.92)	43	NAbs 0 ST (25)	12 (48)	0.89 (0.52-1.44)	11
				NAbs ≥1 ST (27)	17 (63)	1.17 (0.78-1.76)	-17
				Breakthrough (17)	7 (41)	0.77 (0.36-1.35)	23
VNR (45)	10 (22)	0.52 (0.26-0.90)	48	VNR (YFV+; 13)	9 (69)	1.29 (0.75-1.99)	-29
				VNR (YFV-; 22)	14 (64)	1.19 (0.77-1.82)	-19
				DENV infected (10)	3 (30)	0.56 (0.15-1.23)	44

RR, risk ratio; CI, confidence interval; VE, vaccine effectiveness calculated as (1 − RR) × 100, where RR represents the relative risk of infection compared with DENV naïve unvaccinated individuals within each study period; UN, unvaccinated; VR, vaccine responder; VNR, vaccine non-responders; NAbs, neutralizing antibodies; ST, DENV serotype; YFV, yellow fever virus. DENV infections were defined serologically (DENV NS1 and/or serotype-specific seroconversion in paired samples) and included both symptomatic and asymptomatic events.

Together, these results showed an association between vaccination and a modest reduction in infection risk during the early post-vaccination period, but this effect was not observed during the second study period.

### Breakthrough DENV infection through T1 reveals antibody responses consistent with prior vaccine priming

We next examined whether antibody responses elicited by natural DENV infection differed according to prior vaccination, consistent with potential vaccine-associated immune priming. To establish a reference for unprimed immunity, we first analyzed unvaccinated children who experienced primary WT DENV infection between baseline and T1 (UN; n=31). These children exhibited modest cumulative neutralizing and ED3-binding antibody responses and limited serotype breadth, consistent with a primary DENV infection (**Figures 3A–D**).

**Figure 3:**
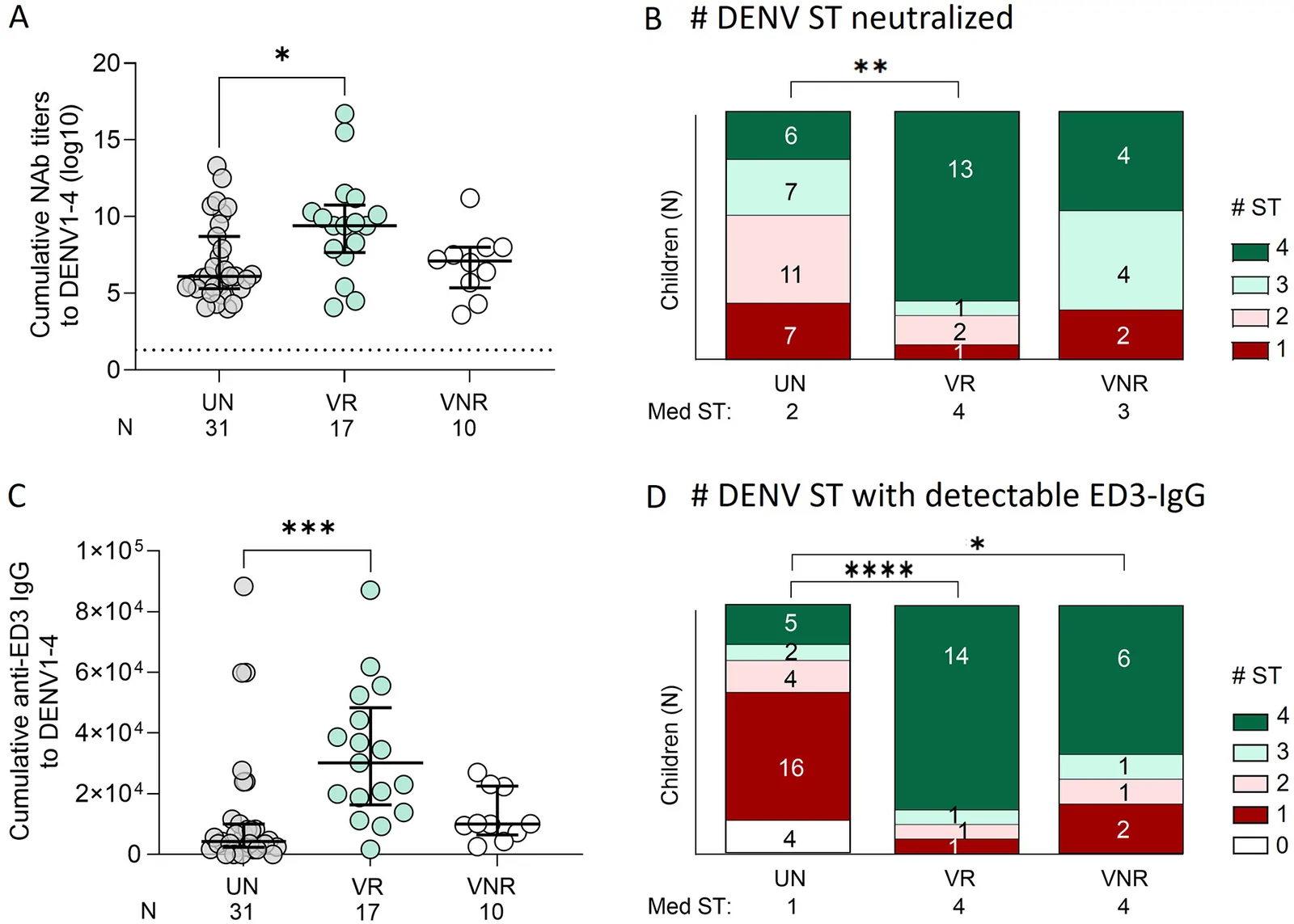
Antibody magnitude and serotype breadth following WT DENV infection occurring during the first study period (BL-T1). Analyses are restricted to children with serologically confirmed WT DENV infection during the BL-T1 interval, corresponding to the infected subgroups summarized in **Table 2**. **(A, C)** Cumulative Ab magnitude. Cumulative NAbs **(A)** and ED3-binding IgG **(C)** responses to DENV1–4 following infection between baseline and T1 are shown for unvaccinated children (UN), vaccine responders with breakthrough infection (VR), and vaccine non-responders with subsequent DENV infection (VNR). Data are shown as median ± interquartile range (IQR) with individual values overlaid. For NAbs, titers below the positivity threshold (<20) were assigned a value of 5 prior to log10 transformation; for ED3 binding, values below the assay threshold (MFI < 1237) were assigned a value of 1. Horizontal dashed lines indicate assay positivity thresholds. Statistical comparisons were performed using the Kruskal-Wallis test with Dunn’s *post hoc* correction. **(B, D)** Serotype breadth of Ab responses. Stacked bars show the number of children neutralizing **(B)** or binding **(D)** to 0, 1, 2, 3, or 4 DENV serotypes following infection between baseline and T1. Serotype breadth reflects the number of DENV serotypes with detectable responses above assay thresholds. Colors indicate increasing serotype breadth from 1 (red) to 4 (green), with numbers inside bars indicating the number of children per category. Median serotype breadth per group is shown below each bar. Pairwise differences in individual-level serotype breadth were assessed using Kruskal-Wallis test with Dunn’s *post hoc* correction.

In contrast, vaccine responders who experienced breakthrough infection (VR; n=17) showed higher cumulative neutralizing antibody titers compared with unvaccinated children (p=0.027), along with broader serotype breadth (defined as the number of DENV serotypes with detectable neutralizing activity per individual; p=0.015). The greater magnitude and breadth of these responses are consistent with vaccine-modulated antibody responses following natural DENV exposure (**Figures 3A–D**).

We next examined vaccine non-responders who experienced DENV infection during the same period (VNR; n=10) to assess whether breakthrough infection could reveal signatures of vaccine-associated immune priming not captured by the Luminex assay at 1:500 serum dilution. In this subgroup, cumulative neutralizing antibody titers following infection did not differ significantly from those observed in either unvaccinated children or vaccine responders. Serotype breadth analysis showed a higher proportion of children neutralizing three to four DENV serotypes (80%) than among unvaccinated children (42%), although this difference did not reach statistical significance (**Figure 3B**).

Cumulative ED3-binding antibody responses followed a similar pattern to cumulative NAbs. Vaccine responders exhibited markedly higher binding responses than unvaccinated children (p=0.0002), whereas non-responders showed binding magnitudes that did not differ significantly from either group (**Figure 3C**). Notably, binding serotype breadth was greater in both vaccinated groups compared with unvaccinated children (p<0.0001 and p=0.019, respectively; **Figure 3D**), suggesting that broader binding antibody responses across serotypes may capture vaccine-associated differences in non-responders that were less evident in neutralizing antibody responses. Effect sizes with 95% CIs supported these patterns and are provided in **Supplementary Table 1**.

DENV2 was the most frequently inferred infecting serotype in both unvaccinated children (18/31) and vaccine responders (7/17) (**Supplementary Table 2**). In an exploratory sensitivity analysis restricted to children with inferred DENV2 infection (UN, n=18; VR, n=7), the pattern of greater post-infection antibody responses among vaccine responders was retained across neutralizing and ED3-binding antibody magnitude and serotype breadth (**Supplementary Table 2**).

Together, these findings are consistent with prior vaccination shaping the magnitude and breadth of antibody responses elicited by subsequent DENV infection, with the clearest differences observed among vaccine responders.

### Vaccine-associated differences in post-infection antibody responses were no longer observed during the second study period (T1–T2)

To determine whether vaccine-associated differences in post-infection antibody responses persisted at later time points, we next examined WT DENV infections occurring during the second study period (T1–T2), corresponding to approximately month 20 and month 40 of follow-up. This interval represents a substantially longer gap between vaccination and infection compared with the first study period.

Following WT DENV infection during T1–T2, no statistically significant differences in cumulative NAb titers measured at T2 were detected among UN, VR, and VNR groups (**Figure 4A**).

**Figure 4:**
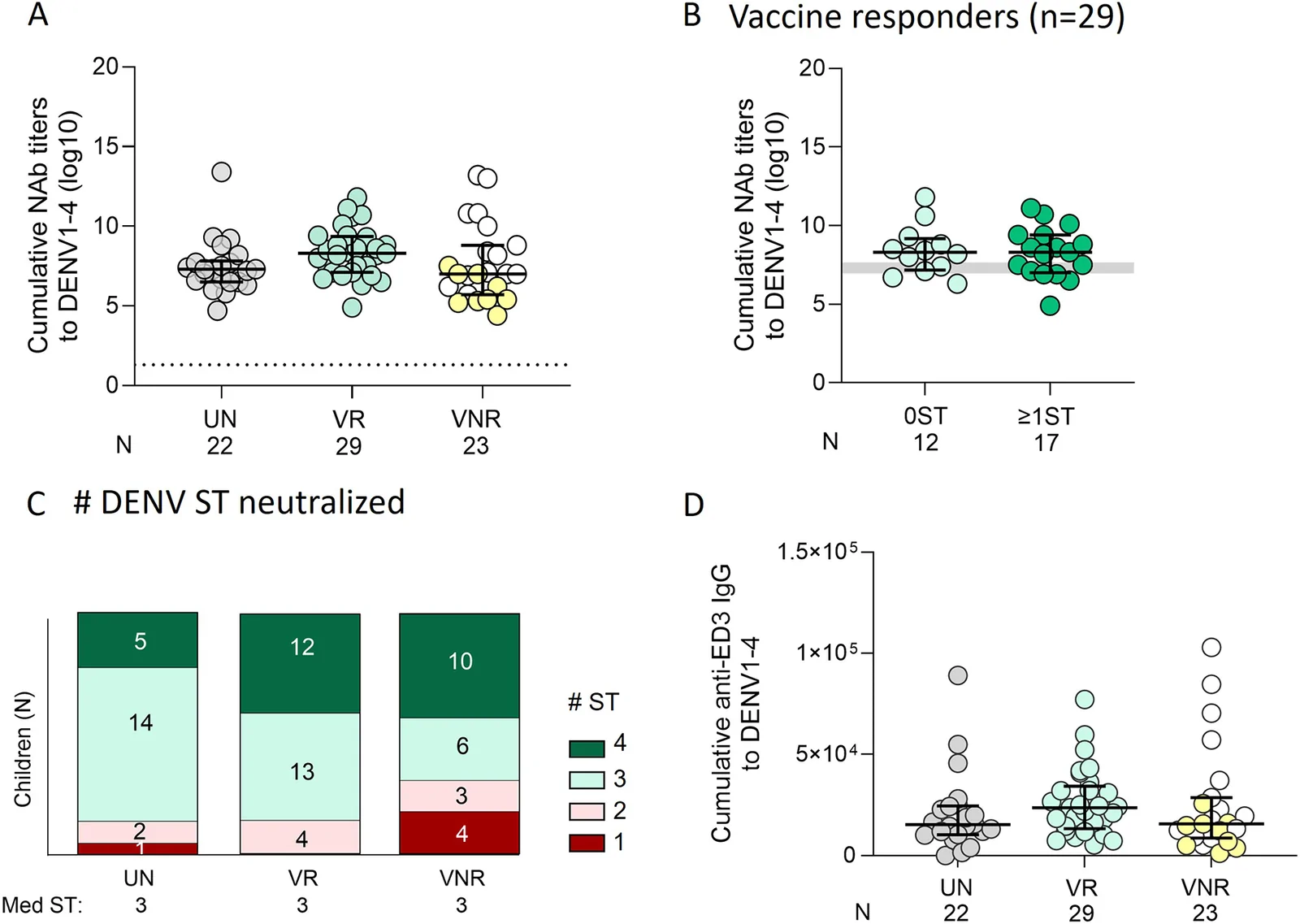
Antibody magnitude and serotype breadth following wild-type (WT) DENV infection during the second study period (T1–T2). **(A)** Cumulative neutralizing antibody (NAb) magnitude at T2 in DENV-infected unvaccinated children (UN), vaccine responders with breakthrough infection (VR), and vaccine non-responders with subsequent DENV infection (VNR). Yellow symbols denote individuals within the VNR group with detectable YFV NS1 antibody responses only at a 1:50 serum dilution. Data is shown as median with IQR. Statistical comparisons were performed using the Kruskal-Wallis test with Dunn’s *post-hoc* correction. **(B)** Cumulative NAb titers at T2 compared between vaccine responders with no detectable DENV neutralization (0-ST) and those neutralizing ≥1 DENV serotype prior to infection (T1). A horizontal gray line indicates the median value in unvaccinated children (UN) for reference. Groups were compared using a Mann-Whitney U test. **(C)** Serotype breadth of NAb responses following infection between T1 and T2. Stacked bars indicate the number of children neutralizing 1, 2, 3, or 4 DENV serotypes. Colors represent increasing breadth from 1 (red) to 4 (green), with numbers inside bars indicating the number of children per category. Median serotype breadth per group is shown below each bar. Differences in individual-level serotype breadth were assessed using the Kruskal-Wallis test with Dunn’s *post hoc* correction. **(D)** ED3-binding IgG responses to DENV1–4 at T2 in UN, VR, and VNR groups. For NAbs, titers below the positivity threshold (<20) were assigned a value of 5 prior to log_10_ transformation; for ED3 binding, values below the assay threshold (MFI<1237) were assigned a value of 1. Horizontal dashed lines indicate assay positivity thresholds.

To further assess whether vaccine-induced neutralization at T1 influenced post-infection responses, vaccine responders were stratified into those with detectable NAbs to one or more vaccine DENV serotypes (≥1 serotype) and those without detectable neutralization to any serotype (0 serotypes). Following WT DENV infection between T1 and T2, no statistically significant difference in cumulative NAb responses was detected between these subgroups (**Figure 4B**), providing no evidence in this analysis that pre-infection vaccine-induced neutralizing antibodies were associated with greater post-infection antibody magnitude during the later follow-up period.

We next examined whether vaccination influenced the breadth of the neutralizing response following infection during the second study period. The distribution of neutralization breadth, defined as the number of DENV serotypes neutralized per individual, did not differ significantly among unvaccinated children, vaccine responders, and non-responders (**Figure 4C**). The majority of children in all groups neutralized three to four serotypes following infection.

Finally, we evaluated cumulative ED3-binding IgG responses to DENV1–4 following WT DENV infection during T1–T2. No statistically significant differences in binding antibody magnitude were detected among unvaccinated children, vaccine responders, and non-responders (**Figure 4D**).

Collectively, these data indicate that the differences in antibody magnitude and breadth observed between vaccinated and unvaccinated children following infection during the first study period were no longer detectable during the second study period.

## Discussion

This longitudinal study provides insight into vaccine responsiveness, the association between a single dose of Dengvaxia^®^ and reduced risk of WT DENV infection, and antibody responses following breakthrough infection in dengue-naïve children.

One of the principal findings of this study is that antibody responses following a single dose of Dengvaxia^®^ in dengue-naïve children were more heterogeneous than captured by the original binary responder/non-responder classification. Low-level YFV NS1 antibody responses were detected in a subset of children originally classified as non-responders, revealing additional heterogeneity in YFV NS1 reactivity within this group.

Beyond vaccine responsiveness, this study provides insight into the association between vaccination and susceptibility to WT DENV infection over time. Previous evaluations of Dengvaxia^®^ have primarily focused on protection against symptomatic and severe dengue disease (20, 21). In contrast, asymptomatic and subclinical infections account for a substantial proportion of DENV infections, contribute to viral transmission, and shape population immunity in endemic settings (22–24).

During the first study period (BL–T1), vaccination was associated with reduced risk of WT DENV infection compared with unvaccinated children. By the second study period (T1–T2), this association was no longer evident. This temporal pattern is consistent with previous reports documenting declines in CYD-TDV–induced antibody responses over time in dengue-endemic populations (25). Although the parent cohort collected demographic, socioeconomic, and other relevant characteristics at enrollment (9), residual confounding cannot be excluded given the observational design and the subset analyzed in the present study.

In contrast, children with prior natural DENV infection had a lower risk of subsequent infection during the second study period, highlighting differences in the observed associations following vaccination versus natural infection. Notably, breakthrough infection among vaccinated children during the first study period was not associated with a lower risk of subsequent reinfection. This observation should be interpreted cautiously given the limited number of breakthrough infections and the possibility that children experiencing early breakthrough infection represent a selected subset of vaccinees with distinct underlying immune responses.

The reduced risk of infection observed during the first study period prompted further evaluation of whether vaccination was associated with differences in the antibody response generated following WT DENV infection. During this period, vaccine responders experiencing breakthrough WT DENV infection showed higher cumulative neutralizing and binding antibody responses and broader serotype breadth compared with unvaccinated children experiencing primary infection, consistent with vaccine-associated immune priming following natural DENV exposure. DENV2 was the most frequently inferred infecting serotype in both vaccine responders and unvaccinated children, and the pattern of greater post-infection antibody responses among vaccine responders was retained in the exploratory analysis restricted to DENV2 infections, suggesting that these differences were not solely attributable to the infecting serotype. Across analyses, serotype breadth revealed vaccine-associated differences that were not always evident when considering antibody magnitude alone.

Importantly, by the second study period (T1–T2), differences in cumulative neutralizing and binding antibody responses following WT DENV infection were no longer evident between vaccinated and unvaccinated children, regardless of pre-infection neutralization status or serotype breadth, paralleling the absence of a detectable difference in infection risk during the same period.

Several limitations should be considered when interpreting these findings. First, serological measurements cannot directly establish the cellular origin of post-infection antibody responses or persistence of vaccine-induced immune memory. The absence of detectable differences between groups during the second study period should therefore not be interpreted as evidence of complete loss of vaccine-induced immune memory, which cannot be established from the available serological data. Longitudinal studies incorporating direct cellular analyses will be needed to address these mechanisms. Second, the precise timing of WT DENV infection relative to annual sample collection was unknown. Thus, the interval between infection and sample collection likely varied among children, and differences in time since infection could have influenced the magnitude and breadth of post-infection antibody responses. In addition, the modest difference in T1 sampling time between vaccinated and unvaccinated children may have influenced comparisons of antibody responses at this time point. Third, the relatively small number of breakthrough infections, particularly among vaccine non-responders, limited the statistical power and precision of subgroup comparisons. Moreover, because vaccination was associated with a reduced risk of WT DENV infection during the first follow-up period, children who experienced breakthrough infection represent a selected subset of vaccinated individuals and may not fully reflect immune responses across the broader vaccinated population. Therefore, while breakthrough infections provide an opportunity to evaluate vaccine-associated post-infection antibody responses, these findings should not be interpreted as representative of all vaccine recipients. Finally, the study evaluated responses following a single-dose vaccination schedule and may not reflect immune dynamics following alternative dosing strategies.

Together, these findings suggest that a single dose of Dengvaxia^®^ in dengue-naïve children was associated with reduced risk of WT DENV infection during the first follow-up period and with higher and broader antibody responses following breakthrough infection during the first follow-up period, but these vaccine-associated differences were not sustained during the second period. In contrast, prior natural infection was associated with a lower risk of subsequent infection during the second study period. Collectively, our findings emphasize the importance of considering both antibody magnitude and serotype breadth when characterizing dengue vaccine-associated immunity over time.

## Data Availability

The raw data supporting the conclusions of this article will be made available by the authors, without undue reservation.

## Supplementary material

Supplementary Table 1 Effect-size estimates for post-infection antibody responses following WT DENV infection during the first study period (BL–T1).

Supplementary Table 2 Inferred infecting serotypes and DENV2-restricted antibody responses following WT DENV infection during the first study period (BL–T1). (A) Distribution of inferred infecting DENV serotypes. (B) Exploratory sensitivity analysis among children with inferred DENV2 infection.

## References

[1] Narvaez F Gutierrez G Pérez MA Elizondo D Nuñez A Balmaseda A . Evaluation of the traditional and revised WHO classifications of dengue disease severity. PloS NeglTrop Dis. (2011) 5:e1397. doi: 10.1371/journal.pntd.0001397 22087348 PMC3210746

[2] Guzman MG Halstead SB Artsob H Buchy P Farrar J Gubler DJ . Dengue: a continuing global threat. Nat Rev Microbiol. (2010) 8:S7–16. doi: 10.1038/nrmicro2460 21079655 PMC4333201

[3] Teo A Tan HD Loy T Chia PY Chua CLL . Understanding antibody-dependent enhancement in dengue: Are afucosylated IgG1s a concern? PloS Pathog. (2023) 19:e1011223. doi: 10.1371/journal.ppat.1011223 36996026 PMC10062565

[4] Capeding RZ Luna IA Bomasang E Lupisan S Lang J Forrat R . Live-attenuated, tetravalent dengue vaccine in children, adolescents and adults in a dengue endemic country: randomized controlled phase I trial in the Philippines. Vaccine. (2011) 29:3863–72. doi: 10.1016/j.vaccine.2011.03.057 21477675

[5] Sridhar S Luedtke A Langevin E Zhu M Bonaparte M Machabert T . Effect of dengue serostatus on dengue vaccine safety and efficacy. N Engl J Med. (2018) 379:327–40. doi: 10.1056/NEJMoa1800820 29897841

[6] Thomas SJ Yoon IK . A review of Dengvaxia®: development to deployment. Hum Vaccin Immunother. (2019) 15:2295–314. doi: 10.1080/21645515.2019.1658503 31589551 PMC6816420

[7] Henein S Swanstrom J Byers AM Moser JM Shaik SF Bonaparte M . Dissecting antibodies induced by a chimeric yellow fever-dengue, live-attenuated, tetravalent dengue vaccine (CYD-TDV) in naive and dengue-exposed individuals. J Infect Dis. (2017) 215:351–8. doi: 10.1093/infdis/jiw576 27932620 PMC6392503

[8] Halstead SB Katzelnick LC Russell PK Markoff L Aguiar M Dans LR . Ethics of a partially effective dengue vaccine: Lessons from the Philippines. Vaccine. (2020) 38:5572–6. doi: 10.1016/j.vaccine.2020.06.079 32654899 PMC7347470

[9] Ylade M Crisostomo MV Daag JV Agrupis KA Cuachin AM Sy AK . Effect of single-dose, live, attenuated dengue vaccine in children with or without previous dengue on risk of subsequent, virologically confirmed dengue in Cebu, the Philippines: a longitudinal, prospective, population-based cohort study. Lancet Infect Dis. (2024) 24:737–45. doi: 10.1016/S1473-3099(24)00099-9 38527474 PMC11187693

[10] White LJ Hein LD Abad Fernandez M Adams C Adams E Freeman E . Safety of a tetravalent live dengue virus vaccine in children responding to one serotype only. JCI Insight. (2026) 11:e200741. doi: 10.1172/jci.insight.200741 41842965 PMC13135402

[11] Sun T Wang Y Song X Li R Mei F Yang M . Impaired humoral immunity identified in inactivated SARS-CoV-2 vaccine recipients without anti-spike RBD antibodies. Microbiol Spectr. (2023) 11:e0278322. doi: 10.1128/spectrum.02783-22 36916970 PMC10101072

[12] Yan K Cai W Cao F Sun H Chen S Xu R . Genetic effects have a dominant role on poor responses to infant vaccination to hepatitis B virus. J Hum Genet. (2013) 58:293–7. doi: 10.1038/jhg.2013.18 23486340

[13] Mantus G Nyhoff LE Edara VV Zarnitsyna VI Ciric CR Flowers MW . Pre-existing SARS-CoV-2 immunity influences potency, breadth, and durability of the humoral response to SARS-CoV-2 vaccination. Cell Rep Med. (2022) 3:100603. doi: 10.1016/j.xcrm.2022.100603 35480625 PMC8960152

[14] Sasaki S He XS Holmes TH Dekker CL Kemble GW Arvin AM . Influence of prior influenza vaccination on antibody and B-cell responses. PloS One. (2008) 3:e2975. doi: 10.1371/journal.pone.0002975 18714352 PMC2500171

[15] Bauer T Jilg W . Hepatitis B surface antigen-specific T and B cell memory in individuals who had lost protective antibodies after hepatitis B vaccination. Vaccine. (2006) 24:572–7. doi: 10.1016/j.vaccine.2005.08.058 16171909

[16] Lopez AL Adams C Ylade M Jadi R Daag JV Molloy CT . Determining dengue virus serostatus by indirect IgG ELISA compared with focus reduction neutralization test in children in Cebu, Philippines: a prospective population-based study. Lancet Glob Health. (2021) 9:e44–51. doi: 10.1016/S2214-109X(20)30392-2 33212030 PMC9358663

[17] Mukherjee S Sirohi D Dowd KA Chen Z Diamond MS Kuhn RJ . Enhancing dengue virus maturation using a stable furin over-expressing cell line. Virology. (2016) 497:33–40. doi: 10.1016/j.virol.2016.06.022 27420797 PMC5502530

[18] Tse LV Meganck RM Dong S Adams LE White LJ Mallory ML . Generation of mature DENVs via genetic modification and directed evolution. mBio. (2022) 13:e0038622. doi: 10.1128/mbio.00386-22 35481749 PMC9239201

[19] Hein LD Castillo IN Medina FA Vila F Segovia-Chumbez B Muñoz-Jordán JL . Multiplex sample-sparing assay for detecting type-specific antibodies to Zika and dengue viruses: an assay development and validation study. Lancet Microbe. (2025) 6:100951. doi: 10.1016/j.lanmic.2024.07.014 39730005 PMC12244352

[20] Hadinegoro SR Arredondo-García JL Capeding MR Deseda C Chotpitayasunondh T Dietze R . Efficacy and long-term safety of a dengue vaccine in regions of endemic disease. N Engl J Med. (2015) 373:1195–206. doi: 10.1056/NEJMoa1506223 26214039

[21] Naderian R Eslami M Ahmad S Paraandavaji E Yaghmayee S Soltanipur M . Efficacy, immune response, and safety of dengue vaccines in adolescents: A systematic review. Rev Med Virol. (2025) 35:e70035. doi: 10.1002/rmv.70035 40195054

[22] Endy TP Yoon IK Mammen MP . Prospective cohort studies of dengue viral transmission and severity of disease. Curr Top Microbiol Immunol. (2010) 338:1–13. doi: 10.1007/978-3-642-02215-9_1 19802574

[23] Gordon A Kuan G Mercado JC Gresh L Avilés W Balmaseda A . The Nicaraguan pediatric dengue cohort study: incidence of inapparent and symptomatic dengue virus infections, 2004-2010. PloS NeglTrop Dis. (2013) 7:e2462. doi: 10.1371/journal.pntd.0002462 24086788 PMC3784501

[24] Salje H Cummings DAT Rodriguez-Barraquer I Katzelnick LC Lessler J Klungthong C . Reconstruction of antibody dynamics and infection histories to evaluate dengue risk. Nature. (2018) 557:719–23. doi: 10.1038/s41586-018-0157-4 29795354 PMC6064976

[25] Velumani S Toh YX Balasingam S Archuleta S Leo YS Gan VC . Low antibody titers 5 years after vaccination with the CYD-TDV dengue vaccine in both pre-immune and naïve vaccinees. Hum Vaccin Immunother. (2016) 12:1265–73. doi: 10.1080/21645515.2015.1126012 26889737 PMC4963049

