# Supplementary material for: A single dose of Dengvaxia is associated with transient reduction in dengue infection risk among dengue-naïve children
Source: Front Immunol. 2026 Oct 14;17:1959704. doi: 10.3389/fimmu.2026.1959704 (PMC13713805; doi:10.3389/fimmu.2026.1959704)
Supplement: Supplementary file 1 [file SupplementaryFile1.docx]

Supplementary Table 1. Effect-size estimates for post-infection antibody responses following WT DENV infection during the first study period (BL–T1).

| Ab response | Group comparison | PI (95% CI) |
| --- | --- | --- |
| Cumulative NAb magnitude | VR vs UN | 0.711 (0.525-0.845) |
|  | VNR vs UN | 0.558 (0.343-0.753) |
| NAb serotype breadth | VR vs UN | 0.786 (0.620-0.892) |
|  | VNR vs UN | 0.661 (0.434-0.832) |
| Cumulative ED3 binding IgG | VR vs UN | 0.824 (0.657-0.919) |
|  | VNR vs UN | 0.697 (0.511-0.835) |
| ED3-binding serotype breadth | VR vs UN | 0.869 (0.738-0.940) |
|  | VNR vs UN | 0.781 (0.585-0.900) |

The Mann-Whitney probabilistic index represents the probability that a randomly selected individual from the vaccinated group has a higher response than a randomly selected individual from the unvaccinated group. A value of 0.5 indicates no tendency toward higher values in either group, with ties assigned half-weight. UN (n=31), unvaccinated; VR (n=17), vaccine responder; VNR (n=10), vaccine non-responder; NAb, neutralizing antibody; CI, confidence interval.

Supplementary Table 2. Inferred infecting serotypes and DENV2-restricted antibody responses following WT DENV infection during the first study period (BL–T1).

A. Distribution of inferred infecting DENV serotypes.

| Groups (n) | DENV1, n | DENV2, n | DENV3, n | DENV4, n | Indeterminate, n |
| --- | --- | --- | --- | --- | --- |
| Unvaccinated (31) | 3 | 18 | 1 | 0 | 9 |
| Vaccine-responders (17) | 0 | 7 | 1 | 0 | 9 |
| Vaccine non-responders (10) | 1 | 4 | 2 | 1 | 2 |

Infecting serotype was inferred from post-infection neutralization profiles based on seroconversion to a single DENV serotype or a ≥4-fold higher neutralizing antibody titer to one serotype relative to the other serotypes. Infections for which no single serotype met either criterion were classified as indeterminate.

B. Exploratory sensitivity analysis among children with inferred DENV2 infection.

| Antibody response | Unvaccinated (18) | Vaccine responders (7) | PI (95% CI) |
| --- | --- | --- | --- |
| Cumulative NAbs,  median (IQR) | 5.55 (5.3-6.1) | 9.40 (6.40–9.50) | 0.71 (0.43–0.95) |
| NAb ST breadth,  median (range) | 2 (1-4) | 4 (1–4) | 0.77 (0.51–0.97) |
| Cumulative ED3-binding Ab,  median (IQR) | 3,504 (2,370-7,575) | 30,168 (20,360-39,429) | 0.87 (0.70–1.00) |
| ED3-binding ST breadth,  median (range) | 1 (0–4) | 4 (3–4) | 0.91 (0.79–1.00) |

Data are presented as median (IQR) for antibody magnitude and median (range) for serotype breadth. PI, Mann-Whitney probabilistic index; CI, confidence interval; ED3, envelope domain III; NAb, neutralizing antibody; ST, serotype.
